# A computational efficient optimization of flow shop scheduling problems

**DOI:** 10.1038/s41598-022-04887-8

**Published:** 2022-01-17

**Authors:** Zhongyuan Liang, Peisi Zhong, Mei Liu, Chao Zhang, Zhenyu Zhang

**Affiliations:** 1 Advanced Manufacturing Technology Centre, Shandong University of Science and Technology, Qingdao, 266590 China; 2 College of Mechanical and Electronic Engineering, Shandong University of Science and Technology, Qingdao, 266590 China

**Keywords:** Computational science, Mechanical engineering

## Abstract

Flow shop scheduling problems are NP-hard problems. Heuristic algorithms and evolutionary metaheuristic algorithms are commonly used to solve this kind of problem. Although heuristic algorithms have high solving speed, the solution quality is not good. Evolutionary algorithms make up for this defect in small-scale problems, but the solution performance will deteriorate with the expansion of the problem scale and there will be premature problems. In order to improve the solving accuracy of flow shop scheduling problems, a computational efficient optimization approach combining NEH and niche genetic algorithm (NEH-NGA) is developed. It is strengthened in the following three aspects: NEH algorithm is used to optimize the initial population, three crossover operators are used to enhance the genetic efficiency, and the niche mechanism is used to control the population distribution. A concrete application scheme of the proposed method is introduced. The results of compared with NEH heuristic algorithm and standard genetic algorithm (SGA) evolutionary metaheuristic algorithm after testing on 101 FSP benchmark instances show that the solution accuracy has been significantly improved.

## Introduction

Scheduling is a decision-making process in which re-sources are allocated to different tasks under certain constraints. Early work in the field of scheduling was driven by manufacturing, and although considerable progress has been made on scheduling problems in many non-manufacturing fields, manufacturing terminology is still use. Resources are usually called machines, and tasks are called jobs, and sometimes jobs may be composed of several basic tasks linked by sequence constraints, called operations. The terms “machine”, “job” and “operation” in scheduling problems are abstract concepts that can represent a wide range of real objects.

Flow shop scheduling problem (FSP), which is a typical combinatorial optimization problem and exists widely in production system and service system. It belongs to NP-hard problem category^1^. Therefore, the study of this problem has important theoretical significance and engineering value, and it is also the most widely studied type of typical scheduling problem. Most of the early researches on scheduling problems use mathematical methods such as integer programming, branch and bound, etc., focusing on theory and trying to get the exact optimal solution.

The flow manufacturing model rises and the process of products becomes more complicated in sync with the wide application of automation in industry. e.g., a FSP with 20 jobs, its solution space is 20!, i.e., 2.4 × 10^18^. Due to the limitation of precise methods in such large-scale problems, a large number of heuristic methods have been widely used, e.g., Gupta, Johnson, Palmer, NEH, RA. These algorithms generate solution based on problem-specific experience and construction rules, which may not get the optimal operation sequence, but can guarantee the local optimality of the processing sequence to a certain extent. Studies show that NEH^2^ proposed by Nawaz, Enscore and Ham in 1983 is the best heuristic algorithm to solve this problem^3–5^. In view of the superiority of NEH algorithm in solving FSP and the deficiency of heuristic algorithm, researchers proposed many extensions of the NEH. Pawel et al.^3^ proposed a new priority order combined with a simple tie-breaking method named NEHNM. Victor Fernandez et al.^6^ proposed a new tie-breaking mechanism based on an estimation of the idle times of the different subsequence with the same best partial makespan to so remedy defects in NEH. LR-NEH(x) method proposed by Pan et al.^6^ represents a good trade-off between CPU time and quality. Fernando et al.^7^ improved the algorithm based on LR-NEH(x) and the new method provided high-quality solutions with computational efficiency, significantly outperforming the best simple heuristics. Through statistics, we find that these variations focus on construction criteria of input sequences for construction phase and the tie-breaking mechanisms of candidate sequences.

The emergence of metaheuristic algorithms has provided more efficient solutions to NP-hard problems. In particular, population evolution intelligence algorithms excel in solving black-box problems, non-integrable and non-differentiable problems. Such as genetic algorithm (GA)^8^, particle swarm optimization (PSO)^9^, ant colony optimization (ACO)^10^, differential evolution algorithm (DE)^11^, etc. Then algorithms based on the characteristics of biological populations such as artificial bee colony (ABC)^12^, firefly algorithm (FA)^13^, cuckoo search (CS)^14^, grey wolf optimizer (GWO)^15^, etc. emerged one after another. Abdel-Basset et al.^16^ proposed a new algorithm that integrates the whale optimization algorithm (WOA) with a local search strategy for tackling the permutation flow shop scheduling problem. Marichelvam et al.^17,18^ proposed a sub-population based hybrid monkey search algorithm and an improved hybrid cuckoo search algorithm to solve the flow shop scheduling problem. The two algorithms have been implemented for some benchmark problems in the literature and the results outperform many other heuristics. Li et al.^19^ analysed the properties of flow shop scheduling problems to minimise maximum completion time, and generate a new dominance rule that is complementary to Szwarc’s rule. In addition, Li et al.^20^ also considered the cost of total time occupied by machines in flow shop scheduling and took it as the optimization objective, proposed a balance method with the optimization objective of makespan, and applied this model to the actual situation of doctors' treatment, and achieved good results. Considering cost active adoption of dynamic scheduling and predictive scheduling is also an extension of FSP when machine is failure and maintenance during the production, and some achievements have been made in the related literature^21–23^ research.

The commonality of these algorithms is that they all require individual encoding and decoding processes, and the difference is the rules for updating the population. These algorithms were initially tested on mathematical functions rather than combinatorial optimization problems. Reeves^24^ firstly showed the feasibility of using GA for such problems by producing a working algorithm. From then on, GA has become one of the most popular algorithms for job shop scheduling problems because of its simplicity, versatility and good robustness. Çalis et al.^25^ statistically shows the algorithms adopted by researchers for job shop scheduling solutions in the period 1997–2012, where GA ranked first with 26.4% of the total. Salido et al.^26^ developed GA to solve an extended version of the job shop scheduling problem in which machines can consume different amounts of energy to process tasks at different rates. Azadeh et al.^27^ presented an integrated simulation and GA for optimum operator allocation in a large multi-product assembly shop. Liang et al.^28^ studied a hybrid algorithm based on GA and SA to solve complex multiproduct scheduling problem with 0-wait constraint. Costa et al.^29^ proposed a hybrid metaheuristic procedure integrating features from genetic algorithm and random sampling search method to effectively cope with FSP. Hamdi et al.^30^ proposed 6 versions of the genetic algorithms based on different genetic operators to minimize the makespan in a two-machine cross-docking FSP. Praveen et al.^31^ proposed a GA approach to minimize the makespan for two batch processing machines in a flow shop and experimental study indicated that the GA approach outperforms the other approaches by reporting better solution. Pavol et al.^32^ proposed a hybrid improvement heuristic for FSP based on the idea of GA and heuristic method. Through statistics, we find that these variations focus on operator adaptation for special scheduling problems, optimization of initial populations and updating criteria of genetic operators. GA applied to job shop scheduling problem follows the standard process of GA for solving general problems, i.e., designing the chromosome encoding and decoding method and forming the initial population, and then evolving the population by selection crossover variants.

Due to the importance and representativeness of FSP in the scheduling field, researchers designed several benchmarks to test and compare the optimization performance of different methods. Carlier^33^ designed 8 benchmarks of different scales, named Car01–Car08. Heller^34^ gave 2 benchmarks named Hel01 and Hel02. Reeves^24^ gave 21 benchmarks named Rec01(odd-numbered)-Rec41. Taillard^35^ proposed 120 benchmarks named Ta001-Ta120 and divided them into 12 groups based on their sizes. The sizes of these problems were greater than that of the rare examples published and correspond to real dimensions of industrial problems. Eva et al.^36^ developed a website for researchers to share a series of examples, which is really a boon in combinatorial optimization and the above benchmarks all can be found on it.

Synthesize the above analysis, heuristic algorithms have high solving speed, the solution quality is not good. Evolutionary algorithms make up for this defect in small-scale problems, but the solution performance will deteriorate with the expansion of the problem scale and there will be premature problems. In order to improve the solving accuracy of FSP problems, a computational efficient optimization approach combining NEH and niche genetic algorithm (NEH-NGA) is developed after we studied the solution space distribution of FSPs. It is strengthened in the following three aspects: NEH algorithm is used to optimize the initial population, three crossover operators are used to enhance the genetic efficiency, and the niche mechanism is used to control the population distribution.

## FSP description and related basic research

### FSP description

There are *n* jobs {*J*_1_, *J*_2_, …, *J*_n_} that have to processed in the same sequence on *m* unrelated machines {*M*_1_, *M*_2_, …, *M*_m_}. Typically, job *J*_i_ contains *m* operations {*O*_i1_, *O*_i2_, …, *O*_im_} in a fixed sequence. The processing time of *J*_i_ on machine is given by {*p*_i1_, *p*_i2_, …, *p*_im_}. The FSP has the following constraints:

1. Every job has to be processed on the fixed machine order.
2. Each operation cannot be repeated processing.
3. Each operation can only be processed by one machine at a time and each machine can only process one operation at a time.
4. All machines are idle at time 0.

It is clear that each operation may wait before being processed if the machine is in processing. The problem consists of minimizing the time between the beginning of the execution of the first job on the first machine and the completion of the execution of the last job on the last machine, and this time is called makespan^5^.

### Analysis of FSP solution space

**Figure 1:**
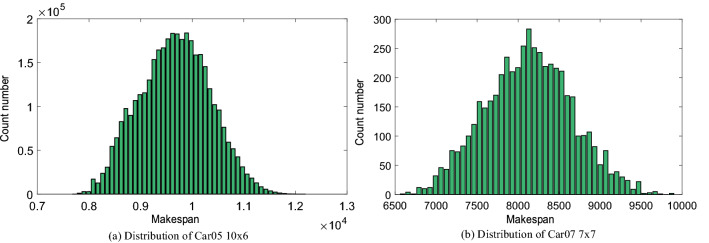
Distribution of instances.

According to the above statement of FSP, we know that the number of solutions for any problem with scale *n*x*m* is *n*!. In order to study the distribution of FSP solutions, we enumerated all the solutions of “Car05” and “Car07”. They’re on the scale of 10 × 6 and 7 × 7 with 3,628,800 and 5040 different job sequences respectively. Figure 1 shows the makespan distribution of “Car05” and “Car07”. For “Car05”, specifically, the number of individuals in [9500, 10500] is 1,751,048, the number of optimal job sequences (job sequences are *J*_5_-*J*_4_-*J*_2_-*J*_1_-*J*_3_-*J*_8_-*J*_6_-*J*_10_-*J*_9_-*J*_7_, *J*_5_-*J*_2_-*J*_4_-*J*_1_-*J*_3_-*J*_8_-*J*_6_-*J*_10_-*J*_9_-*J*_7_, *J*_4_-*J*_5_-*J*_2_-*J*_1_-*J*_3_-*J*_8_-*J*_6_-*J*_10_-*J*_9_-*J*_7_ and their makespan = 7720) is 3 and the worst (job sequence are *J*_10_-*J*_8_-*J*_7_-*J*_1_-*J*_9_-*J*_5_-*J*_3_ -*J*_4_-*J*_2_-*J*_6_, *J*_10_-*J*_8_-*J*_7_-*J*_1_-*J*_9_-*J*_3_-*J*_5_-*J*_4_-*J*_2_-*J*_6_, *J*_10_-*J*_7_-*J*_8_-*J*_1_-*J*_9_-*J*_5_-*J*_3_-*J*_4_-*J*_2_-*J*_6_, *J*_10_-*J*_7_-*J*_8_-*J*_1_-*J*_9_-*J*_3_-*J*_5_-*J*_4_-*J*_2_-*J*_6_ and the makespan = 12,152) is 4. It took 5277 s to evaluate all the solutions on computer. This indicates that enumerations for larger FSP problems will take more time, even if the scale changes from 10 to 11 jobs, the time will increase by 11 times. For “Car07, specifically, the number of individuals in [7500, 9000] is 4145, the number of optimal job sequences (job sequences are *J*_5_-*J*_4_-*J*_2_-*J*_6_-*J*_7_-*J*_3_-*J*_1_ and the makespan = 6590) is 1 and the worst (job sequence are *J*_1_-*J*_7_-*J*_5_-*J*_3_-*J*_6_-*J*_2_-*J*_4_, *J*_1_-*J*_5_-*J*_7_-*J*_3_-*J*_6_-*J*_2_-*J*_4_ and the makespan = 9872) is 2.

**Figure 2:**
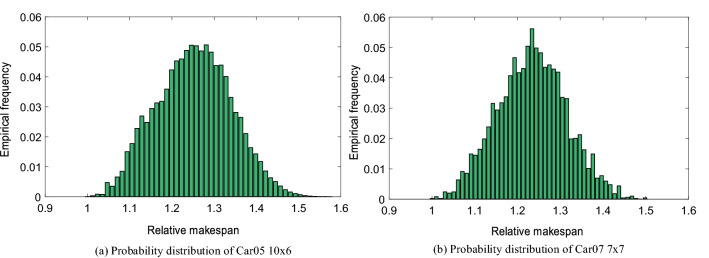
Probability distribution of instances.

Figure 2 shows the relative makespan probability distribution of “Car05” and “Car07”. The relative makespan is makespan divided by optimal makespan, which means 1 represent the optimal solution. According to the distribution, it can be seen that the number of 1% higher than the optimal solution is very small, and it is difficult to find this region by pure random search. The average solution of these two cases is 25.2% and 23.25% higher than the optimal solution respectively. It can also be seen from Fig. 2 that their solutions are more concentrated near this position. This means that the random initial population of the general population evolutionary algorithm is highly likely to be distributed in this interval.

## Methods

Through our preliminary study, it is found that the advantage of heuristic algorithm is the speed of constructing scheduling solutions, but the scheduling quality is not special, among which NEH method has the best performance. Theoretically, the global convergence performance of standard genetic algorithm (SGA) can guarantee the robustness to the initial value in the search process, but it is difficult in the actual time. Sometimes, the effect of large-scale FSP solution is worse than that of NEH, which due to the dependence of the optimization performance and efficiency of the algorithm on the initial population. Therefore, optimizing the initial population through NEH may be a good choice.

In the process of SGA, the structure of crossover operators is always unchanged, which easily leads to the loss of effective features of parents. Therefore, splitting the population into several sub-populations with different crossover operators for evolution may make the algorithm perform well in terms of population diversity and search performance.

In SGA, mating is completely random. In the later stage of evolution, large numbers of individuals concentrate on a local optimum. Without knowing the spatial distribution of solution, we cannot intervene to get rid of local optimum. Niche genetic algorithm is an effective method to solve multimodal optimization. Perhaps we can find the global optimal solution by using the niche formation idea.

**Figure 3:**
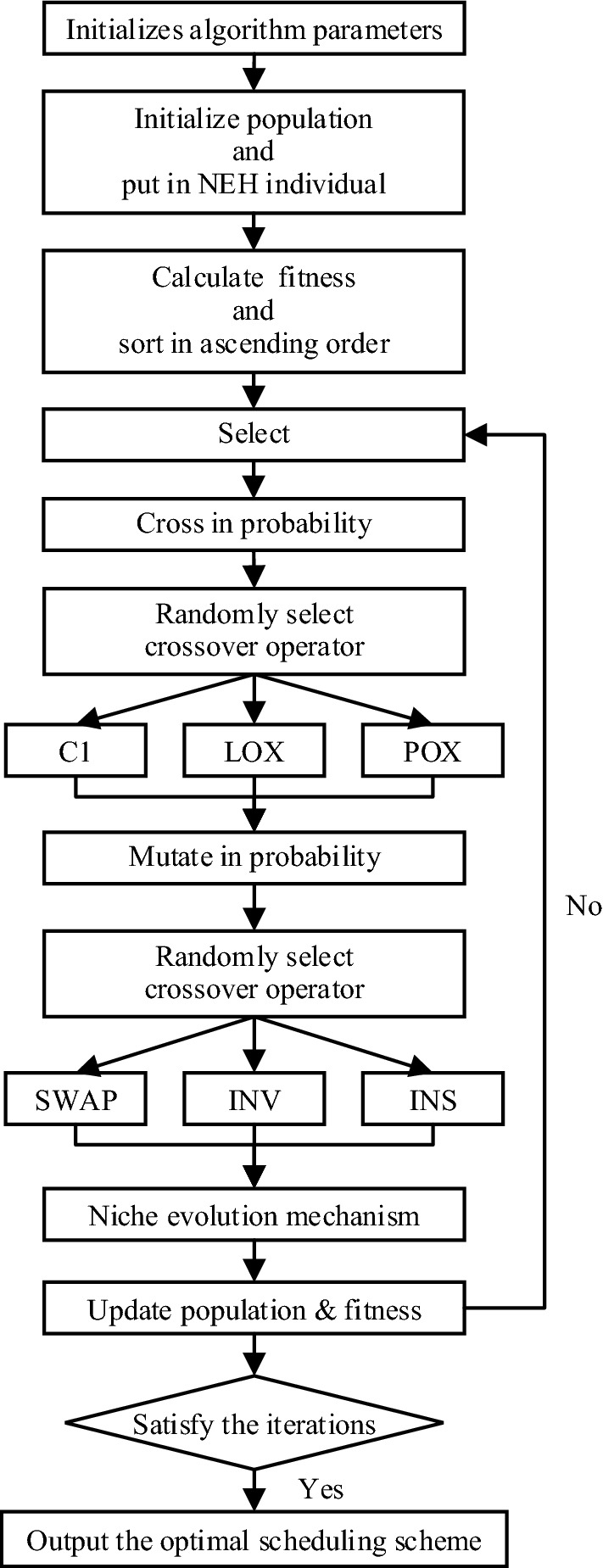
Flow chart for NEH-NGA.

Based on the above analysis, we developed a niche genetic algorithm based on NEH to search for the global optimum of FSP, which is called NEH-NGA for short. The structural framework of NEH-NGA conforms to the general process of SGA. Figure 3 shows the general steps of the proposed algorithm. The following parts of this section will briefly review the basic methods and specific operational details in NEH-NGA. Let’s use the benchmark “Car5” as an example to introduce.

### Encoding and decoding

**Figure 4:**
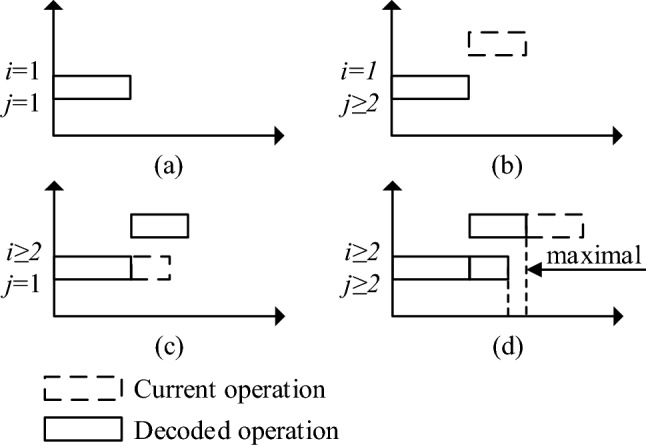
Decoding principle.

Encoding is the primary problem to be solved in the application of GA, and also a key step in the design of GA. In FSP, due to the same order of all jobs, it is possible to simplify the operations within the same job into a whole, that is, encoding based on the job. The serial number of the job is the value of chromosome gene, and the sequence of genes is the processing order of the jobs. This means that in a feasible scheduling solution, the values of all genes are different. E.g., a chromosome “2-1-4-5-7-3-8-10-9-6” is a feasible scheduling solution represents the processing order “*J*_2_-*J*_1_-*J*_4_-*J*_5_-*J*_7_-*J*_3_-*J*_8_-*J*_10_-*J*_9_-*J*_6_”. In essence, decoding is the process of converting chromosomes into scheduling schemes, as well as the process of calculating the start time and end time of each operation. And then the Gantt chart can be got. Whether the target function is makespan or anything else such as machine load, device idle rate, etc., is expanded on this basis. When decoding, our decoding method is shown in Algorithm 1. Decoding follows the principle that no operation can be advanced without changing the processing order on the machine. Figure 4 illustrates the decoding principle.
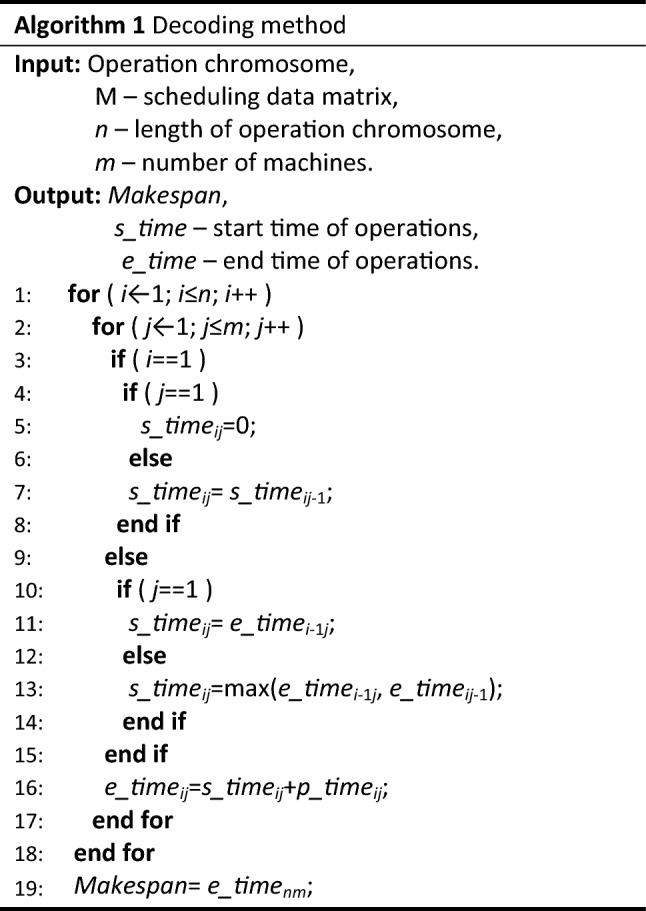

### NEH optimizes the initial population

In SGA, the initial population can be obtained by random sorting of sequence 1 to *n*. On this basis, the quality of the population can be improved by putting the individual obtained by NEH into the initial population. In Introduction, we find that the variations of NEH focus on construction criteria of input sequences for construction phase and the tie-breaking mechanisms of candidate sequences. However, in the proposed algorithm of this paper, population evolution makes the above improved heuristic mechanism irrelevant. Let’s recall NEH method:

- *Step 1* Sort the n jobs in descending order of the sums of processing times on the machines.
- *Step 2* Take the first 2 jobs of the sequences and sort in all possible ways, then the one with a better makespan is selected as the fixed sequence.
- *Step 3* Starting with the 3rd job of the job sequence got in Step 1, insert it into all positions of the fixed sequence, and select the sequence with the best fitness as the new fixed sequence, until all the jobs have been scheduled.
- *Step 4* Output the latest fixed sequence as the best scheduling scheme.

### Selection, crossover and mutation

In GA, the commonly used selection operators are: rotating selection operator, rotating selection operator with ranking, random consistent selection operator and tournament selection operator. In this paper, the tournament selection operator is used. During the selection of the tournament selection operator, *k* individuals are randomly selected from the population, and the one with the best fitness among the k individuals is identified as the optimal individual. This optimal individual is an individual in the next generation population, and the process repeats several times to produce a new population.

In GA, crossover refers to the process in which two mutually paired chromosomes exchange some of their genes with each other in some way, thus forming two new individuals. Crossover operator is the main operator for global search of updating population. Numerical function optimization usually adopts binary encoding, and the parent characteristics can be preserved through single point crossing or multi-point crossing. In combinatorial optimization, the above crossover operator is no longer applicable, and the single crossover operator causes serious loss of effective information of the parent generation, so we adopt three crossover operators to cross.

**Figure 5:**
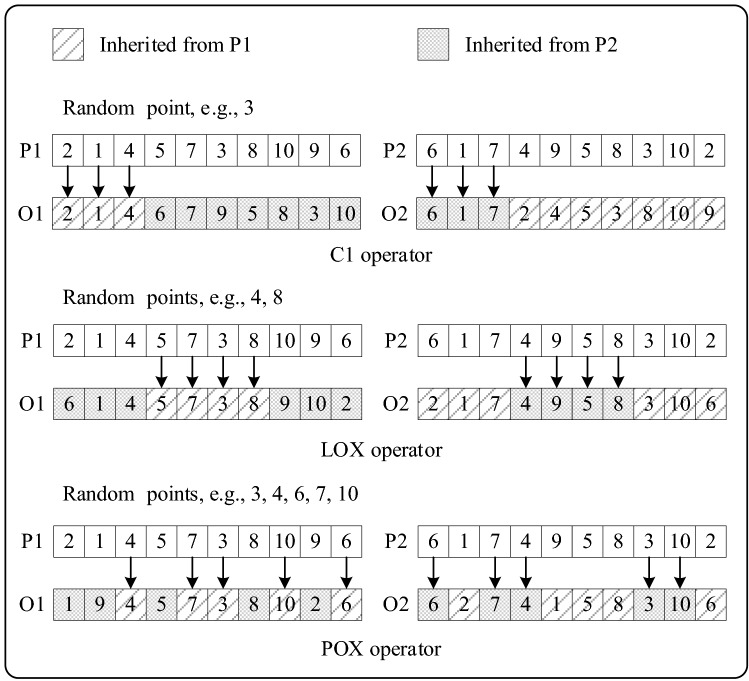
Crossover operators.

Reeves^24^ proposed two crossover operators “C1” and “C2” when he first used GA to solve FSP and “C2” was expected that it would disrupt the chromosome much more than “C1”. As shown in Fig. 5, this crossover operator is similar to one-point crossover operator. A random crossover site is generated and the gene fragments preceding the crossover site are preserved in situ. Genes identical to the above gene fragments are deleted from both paternal chromosomes, and the remaining genes are sequentially passed on to the other offspring chromosomes.

Falkenauer et al.^37^ proposed a linear order crossover (LOX) operator and it can be thought of as a variation of two-point crossover operator. Specifically, as shown in Fig. 5, two random crossover sites are determined, and the gene fragments between the two sites are preserved in situ. Genes identical to the above gene fragments are deleted from both paternal chromosomes, and the remaining genes are sequentially passed on to the other offspring chromosomes.

Kacem^38^ proposed a position-based crossover (POX) operator and it can be thought of as a variation of multi-point crossover operator. This paper is no longer crossing based on position but on the job numbers. Specifically, as shown in Fig. 5, a random set of job numbers is generated, and the genes represented by the job numbers in the above set are preserved in situ. Genes identical to the above genes are deleted from both paternal chromosomes, and the remaining genes are sequentially passed on to the other offspring chromosomes.

Wang^39^ compared the effects of the above three crossover operators in SGA and the results show that there is no significant difference in search quality among them. Considering that the reservation of valid genes by a single crossover operator is one-sided, this paper uses the above three operators randomly.

When individual fitness no longer evolves and does not reach global optimum, it means that the algorithm enters prematurity. The phenomenon is attributed to the defect of the effective gene, and mutation can increase the population diversity to overcome this condition. The common mutation methods are basically based on two-point mutation. By the random two points, genes in chromosomes can perform swapping mutation, inverse mutation and inserting mutation.

**Figure 6:**
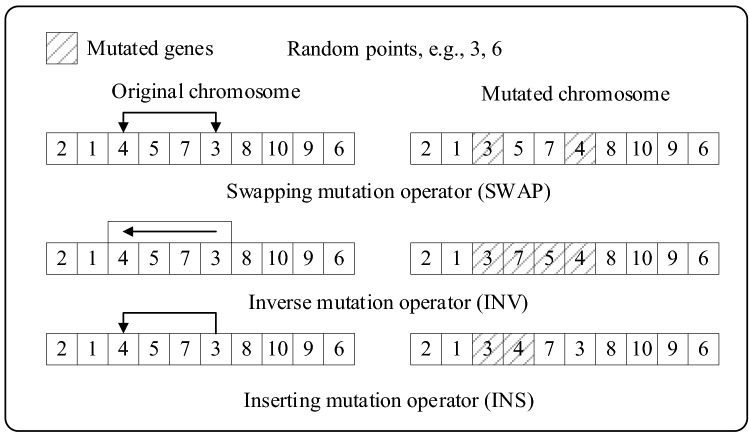
Mutation operators.

Figure 6 shows the principle of the above 3 mutation operators. Swapping mutation is swapping genes at the two points. Inverse mutation is sort genes between the two points in reverse order. Inserting mutation is inserting a gene from one point into the other. A similar integrated approach is to resort the genes between the two points randomly and the above 3 methods are all belong to the integrated method. There are {*k* − (*i* − 1)}! Variation solutions that can be produced by the integrated method, where *i*, *k* are the random mutation points. Since the neighborhood solution space will increase sharply with the increase of the distance between mutation points, the random mixing of the above three mutation operators will be the simplest and most effective mutation means.

### Niche evolution mechanism

“Niche” is a concept derived from biology and it refers to a specific living environment. In the course of their evolution, creatures generally live together with their own species and reproduce together. Refine this idea and applied it to optimization: when the Hamming distance of two individuals is less than a predetermined value (or called niche distance), the individual with the smaller fitness will be penalized.

The idea of niche genetic algorithm (NGA) proposed in this paper is: firstly, the Hamming distance between individuals in the population is compared in pairs. If the Hamming distance is less than the pre-set distance L, then compare the fitness, and the individual with lower fitness will be imposed a strong penalty function to greatly reduce its fitness. In this way, for the two individuals in a single peak range, the poor one’s fitness becomes worse after processing, and the probability of its being eliminated in the subsequent evolutionary process increases. In other words, there will be only one good individual in one single peak range, which not only maintains the diversity of the population, but also keeps a certain distance between individuals. In addition, individuals can be dispersed in the whole solution space, and a niche genetic algorithm is realized. The steps are as follows:

- *Step 1*: Generated u individuals {× 1, × 2,…, × u} randomly to form the initial population *P*, and calculate the fitness *F*(*x*) of each individual.
- *Step 2* Sort individuals in descending order according to their fitness, and record the first *v* (*v* < *u*) individuals in filial-population Q.
- *Step 3* Perform selection, crossover and mutation to population P according to rules and get the updated P.
- *Step 4* Merge *P* and *Q*, and then calculate the Hamming distance *H*(*x*_i_, *x*_j_) between these u + v individuals. If *H*(*x*_i_, *x*_j_) < L, then *min*(*F*(*x*_i_), *F*(*x*_j_)) = Penalty, where Penalty = *Avg*(*P*).
- *Step 5* Update the fitness of these u + v individuals and sort them in descending order. Then select the first u individuals to form new P.
- *Step 6* If the termination condition is not met, then turn Step 2. Otherwise, end loop.

## Results and discussion

### Parameters setting and experimentation

In order to examine the effectiveness of the proposed NEH-NGA, this paper compared NEH-NGA with the NEH Algorithm which is the most popular heuristic in references and SGA which is widely used in combinatorial optimization. In this section, we tested 101 benchmark instances of FSP and the tested sets of benchmarks have been introduced in “Introduction” section. The relevant data and the known optimal solutions are available on the website developed by Eva et al.^36^.

MATLAB 21(a) was used to test benchmark instances of FSP. The CPU frequency of the computer (Intel i5-4460) is 3.20 GHz and the memory is 8 GB. Makespan was regarded as the evaluation index of computational efficient, while in evolutionary algorithms, the number of iterations of the first occurrence of the optimal solution was recorded to regard as the convergence efficiency.

Different setting parameters for different sizes problems. For the reason of optimization is a complex process, parameters need to be obtained through continuous simulation tests. Table 1 shows the specific parameter settings in the proposed algorithm. The classification is based on the results of our many experiments and theoretical support. E.g., for the instance “Car07” with scale “7 × 7”, its solution space is 7! = 5040. We start with a population of 50, and after 50 iterations, we can theoretically search for 2500 different solutions. This coverage is already quite high, and SGA actually finds the optimal solution on average in the fifth generation. For large-scale FSPs, the algorithm can ensure the validity of the parameters we set in the actual solving experiences, although the gap between the total number of searches and the solution space is large.

**Table 1:** Parameters setting.

Parameters	Common problem scales			
*Pc*	0.8	0.8	0.8	0.8
*Pm*	0.1	0.1	0.1	0.1

Parameters	Problem scale with classification 1			
	*n* < 10	10 ≤ *n* < 20	20 ≤ *n* < 50	*n* ≥ 50
*u*	50	100	150	200
*v*	10	20	40	100
*L*	3	5	8	10

Parameters	Problem scale with classification 2			
	*n* × *m* < 100	100 ≤ *n* × *m* < 500	500 ≤ *n* × *m* < 2000	*n* × *m* ≥ 2000
*gen*	50	100	150	200

The parameters in Table 1 are described below:

- *Pc*Probability of crossover, *Pm* Probability of mutation, *U* Number of individuals per generation, *V* Number of individuals of parent population in niche evolution mechanism, *L* Pre-set Hamming distance, *Gen* Number of evolutionary iterations.

For each benchmark instance, it was solved for 20 times to obtain the average result. However, the heuristic NEH algorithm is different from evolutionary algorithms and it can be calculated once. As “Car” series, “Rec” series and “Hel” series contain part of the scale of “Taillard” series, we selected parts of different scales in “Taillard” series for testing.

### Results and discussion

**Figure 7:**
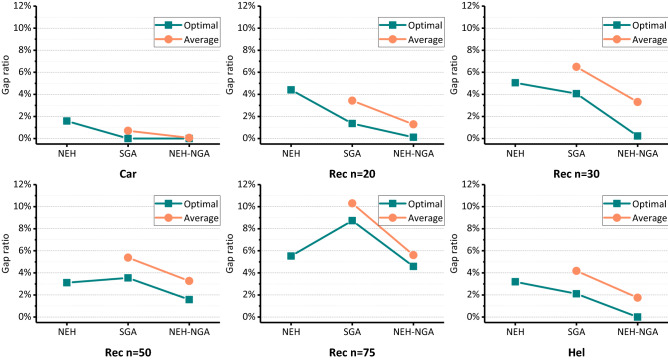
Comparison on Car, Rec and Hel benchmarks.

**Figure 8:**
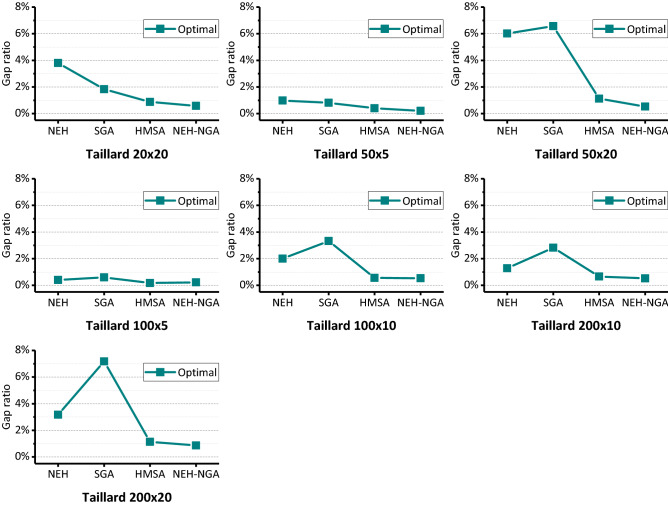
Comparison on Taillard benchmarks.

The post-test statistics are shown in Tables 2 and 3. In Table 2, the solution results of benchmark series “Car”, “Rec” and “Hel” are compared in detail with those of classical algorithms and the solutions on benchmark series “Taillard” are shown in Table 3.*C is the best solution we know^40^. The values of “gap ratio” show the percentage of difference between known optimum and obtained optimum in experience. To facilitate observation, we averaged the data in Tables 2 and 3 according to different problem series and scales, and the results are shown in Figs. 7 and 8.

**Table 2:** Solutions on Car, Rec and Hel benchmarks.

Order	Instance	Scale (nxm)	*C	NEH		SGA				NEH-NGA			
				Optimul	Gap ratio (%)	Optimul	Gap ratio (%)	Average optimum	Average Gap ratio (%)	Optimul	Gap ratio (%)	Average optimum	Average gap ratio (%)
1	Car01	11 × 5	7038	7038	0.00	7038	0.00	7038.00	0.00	7038	0.00	7038.00	0.00
2	Car02	13 × 4	7166	7376	2.93	7166	0.00	7235.20	0.97	7166	0.00	7166.00	0.00
3	Car03	12 × 5	7312	7399	1.19	7312	0.00	7396.85	1.16	7312	0.00	7332.25	0.28
4	Car04	14 × 4	8003	8129	1.57	8003	0.00	8068.10	0.81	8003	0.00	8003.00	0.00
5	Car05	10 × 6	7720	7835	1.49	7720	0.00	7797.35	1.00	7720	0.00	7735.50	0.20
6	Car06	8 × 9	8505	8773	3.15	8505	0.00	8560.40	0.65	8505	0.00	8505.00	0.00
7	Car07	7 × 7	6590	6590	0.00	6590	0.00	6631.65	0.63	6590	0.00	6590.00	0.00
8	Car08	8 × 8	8366	8564	2.37	8366	0.00	8398.50	0.39	8366	0.00	8366.00	0.00
9	Rec01	20 × 5	1247	1320	5.85	1249	0.16	1291.95	3.60	1247	0.00	1254.25	0.58
10	Rec03	20 × 5	1109	1116	0.63	1111	0.18	1130.35	1.93	1109	0.00	1112.88	0.35
11	Rec05	20 × 5	1242	1296	4.35	1245	0.24	1261.05	1.53	1242	0.00	1244.63	0.21
12	Rec07	20 × 10	1566	1626	3.83	1584	1.15	1616.95	3.25	1566	0.00	1581.00	0.96
13	Rec09	20 × 10	1537	1583	2.99	1561	1.56	1594.10	3.72	1537	0.00	1562.13	1.63
14	Rec11	20 × 10	1431	1550	8.32	1473	2.94	1500.00	4.82	1438	0.49	1464.00	2.31
15	Rec13	20 × 15	1930	2002	3.73	1956	1.3	2002.40	3.75	1935	0.26	1963.75	1.75
16	Rec15	20 × 15	1950	2025	3.85	1982	1.64	2016.00	3.38	1950	0.00	1976.38	1.35
17	Rec17	20 × 15	1902	2019	6.15	1959	3.00	1994.80	4.88	1907	0.26	1947.50	2.39
18	Rec19	30 × 10	2093	2185	4.40	2175	3.92	2211.15	5.65	2098	0.24	2154.13	2.92
19	Rec21	30 × 10	2017	2131	5.65	2076	2.93	2130.45	5.62	2022	0.25	2070.25	2.64
20	Rec23	30 × 10	2011	2110	4.92	2070	2.93	2124.25	5.63	2016	0.25	2066.25	2.75
21	Rec25	30 × 15	2513	2644	5.21	2636	4.89	2681.20	6.69	2518	0.20	2605.13	3.67
22	Rec27	30 × 15	2373	2505	5.56	2470	4.09	2522.55	6.30	2378	0.21	2445.50	3.06
23	Rec29	30 × 15	2287	2391	4.55	2415	5.60	2493.65	9.04	2292	0.22	2397.00	4.81
24	Rec31	50 × 10	3045	3171	4.14	3249	6.70	3292.30	8.12	3150	3.45	3225.88	5.94
25	Rec33	50 × 10	3114	3241	4.08	3189	2.41	3265.35	4.86	3149	1.12	3197.38	2.68
26	Rec35	50 × 10	3277	3313	1.10	3327	1.53	3381.15	3.18	3282	0.15	3315.75	1.18
27	Rec37	75 × 20	4951	5284	6.73	5401	9.09	5476.40	10.61	5233	5.70	5282.13	6.69
28	Rec39	75 × 20	5087	5299	4.17	5469	7.51	5549.50	9.09	5299	4.17	5359.88	5.36
29	Rec41	75 × 20	4960	5242	5.69	5436	9.60	5514.80	11.19	5153	3.89	5197.50	4.79
30	Hel01	100 × 10	513	523	1.95	527	2.73	531.90	3.68	513	0.00	522.38	1.83
31	Hel02	20 × 10	135	141	4.44	137	1.48	141.30	4.67	135	0.00	137.25	1.67

**Table 3:** Solutions on taillard benchmarks.

Order	Instance	Scale (nxm)	*C	NEH		SGA		HMSA^17^		NEH-NGA	
				Optimul	Gap ratio (%)	Optimul	Gap ratio (%)	Optimul	Gap ratio (%)	Optimul	Gap ratio (%)
1	Ta021	20 × 20	2297	2410	4.92	2336	1.70	2324	1.18	2297	0.00
2	Ta022	20 × 20	2100	2150	2.38	2144	2.10	2112	0.57	2112	0.57
3	Ta023	20 × 20	2326	2411	3.65	2364	1.63	2348	0.95	2326	0.00
4	Ta024	20 × 20	2223	2264	1.84	2264	1.84	2242	0.85	2264	1.84
5	Ta025	20 × 20	2291	2397	4.63	2330	1.70	2320	1.27	2305	0.61
6	Ta026	20 × 20	2226	2349	5.53	2255	1.30	2249	1.03	2245	0.85
7	Ta027	20 × 20	2273	2383	4.84	2303	1.32	2290	0.75	2290	0.75
8	Ta028	20 × 20	2200	2249	2.23	2249	2.23	2224	1.09	2215	0.68
9	Ta029	20 × 20	2237	2313	3.40	2279	1.88	2246	0.40	2248	0.49
10	Ta030	20 × 20	2178	2277	4.55	2234	2.57	2192	0.64	2178	0.00
11	Ta031	50 × 5	2724	2733	0.33	2735	0.40	2728	0.15	2724	0.00
12	Ta032	50 × 5	2834	2882	1.69	2864	1.06	2846	0.42	2834	0.00
13	Ta033	50 × 5	2621	2640	0.72	2650	1.11	2642	0.80	2630	0.34
14	Ta034	50 × 5	2751	2787	1.31	2778	0.98	2762	0.40	2755	0.15
15	Ta035	50 × 5	2863	2868	0.17	2887	0.84	2866	0.10	2866	0.10
16	Ta036	50 × 5	2829	2840	0.39	2852	0.81	2832	0.11	2829	0.00
17	Ta037	50 × 5	2725	2769	1.61	2746	0.77	2748	0.84	2736	0.40
18	Ta038	50 × 5	2683	2707	0.89	2704	0.78	2690	0.26	2694	0.41
19	Ta039	50 × 5	2552	2617	2.55	2586	1.33	2564	0.47	2558	0.24
20	Ta040	50 × 5	2782	2786	0.14	2782	0.00	2796	0.50	2794	0.43
21	Ta051	50 × 20	3875	4082	5.34	4093	5.63	3896	0.54	3880	0.13
22	Ta052	50 × 20	3715	3921	5.55	3983	7.21	3746	0.83	3738	0.62
23	Ta053	50 × 20	3668	3888	6.00	3911	6.62	3694	0.71	3690	0.60
24	Ta054	50 × 20	3752	3993	6.42	3966	5.70	3814	1.65	3776	0.64
25	Ta055	50 × 20	3635	3835	5.50	3911	7.59	3686	1.40	3673	1.05
26	Ta056	50 × 20	3698	3914	5.84	3896	5.35	3722	0.65	3713	0.41
27	Ta057	50 × 20	3716	3952	6.35	3998	7.59	3766	1.35	3754	1.02
28	Ta058	50 × 20	3709	3938	6.17	3979	7.28	3768	1.59	3709	0.00
29	Ta059	50 × 20	3765	3952	4.97	4000	6.24	3812	1.25	3781	0.42
30	Ta060	50 × 20	3777	4079	8.00	4020	6.43	3826	1.30	3795	0.48
31	Ta061	100 × 5	5493	5519	0.47	5505	0.22	5502	0.16	5505	0.22
32	Ta062	100 × 5	5268	5284	0.30	5290	0.42	5272	0.08	5268	0.00
33	Ta063	100 × 5	5175	5219	0.85	5221	0.89	5192	0.33	5219	0.85
34	Ta064	100 × 5	5014	5037	0.46	5035	0.42	5020	0.12	5014	0.00
35	Ta065	100 × 5	5250	5261	0.21	5280	0.57	5254	0.08	5261	0.21
36	Ta066	100 × 5	5135	5141	0.12	5164	0.56	5144	0.18	5141	0.12
37	Ta067	100 × 5	5246	5266	0.38	5292	0.88	5264	0.34	5252	0.11
38	Ta068	100 × 5	5106	5107	0.02	5137	0.61	5114	0.16	5106	0.00
39	Ta069	100 × 5	5454	5500	0.84	5506	0.95	5466	0.22	5474	0.37
40	Ta070	100 × 5	5328	5346	0.34	5353	0.47	5332	0.08	5346	0.34
41	Ta071	100 × 10	5770	5846	1.32	5955	3.21	5792	0.38	5780	0.17
42	Ta072	100 × 10	5349	5453	1.94	5543	3.63	5368	0.36	5358	0.17
43	Ta073	100 × 10	5677	5781	1.83	5823	2.57	5694	0.30	5700	0.41
44	Ta074	100 × 10	5791	5942	2.61	6056	4.58	5826	0.60	5833	0.73
45	Ta075	100 × 10	5468	5679	3.86	5750	5.16	5514	0.84	5509	0.75
46	Ta076	100 × 10	5303	5375	1.36	5447	2.72	5324	0.40	5319	0.30
47	Ta077	100 × 10	5599	5723	2.21	5747	2.64	5628	0.52	5644	0.80
48	Ta078	100 × 10	5623	5737	2.03	5816	3.43	5664	0.73	5668	0.80
49	Ta079	100 × 10	5875	5983	1.84	6053	3.03	5912	0.63	5896	0.36
50	Ta080	100 × 10	5845	5903	0.99	5978	2.28	5892	0.80	5890	0.77
51	Ta091	200 × 10	10,868	10,942	0.68	11,066	1.82	10,932	0.59	10,968	0.92
52	Ta092	200 × 10	10,494	10,735	2.30	10,885	3.73	10,624	1.24	10,594	0.95
53	Ta093	200 × 10	10,922	11,027	0.96	11,203	2.57	11,006	0.77	10,992	0.64
54	Ta094	200 × 10	10,889	11,057	1.54	11,036	1.35	11,024	1.24	10,984	0.87
55	Ta095	200 × 10	10,524	10,684	1.52	10,846	3.06	10,574	0.48	10,565	0.39
56	Ta096	200 × 10	10,331	10,445	1.10	10,720	3.77	10,369	0.37	10,354	0.22
57	Ta097	200 × 10	10,857	10,966	1.00	11,188	3.05	10,907	0.46	10,876	0.18
58	Ta098	200 × 10	10,731	10,811	0.75	11,032	2.80	10,794	0.59	10,784	0.49
59	Ta099	200 × 10	10,438	10,579	1.35	10,746	2.95	10,482	0.42	10,497	0.57
60	Ta100	200 × 10	10,676	10,844	1.57	11,011	3.14	10,720	0.41	10,676	0.00
61	Ta101	200 × 20	11,294	11,653	3.18	12,119	7.30	11,342	0.43	11,342	0.43
62	Ta102	200 × 20	11,420	11,692	2.38	12,275	7.49	11,584	1.44	11,575	1.36
63	Ta103	200 × 20	11,446	11,852	3.55	12,282	7.30	11,568	1.07	11,461	0.13
64	Ta104	200 × 20	11,347	11,782	3.83	12,222	7.71	11,480	1.17	11,465	1.04
65	Ta105	200 × 20	11,311	11,685	3.31	12,153	7.44	11,452	1.25	11,469	1.40
66	Ta106	200 × 20	11,282	11,629	3.08	12,196	8.10	11,378	0.85	11,386	0.92
67	Ta107	200 × 20	11,456	11,832	3.28	12,332	7.65	11,624	1.47	11,534	0.68
68	Ta108	200 × 20	11,415	11,913	4.36	12,271	7.50	11,613	1.73	11,548	1.17
69	Ta109	200 × 20	11,343	11,698	3.13	12,265	8.13	11,524	1.60	11,511	1.48
70	Ta110	200 × 20	11,422	11,785	3.18	12,385	8.43	11,574	1.33	11,480	0.51

On some small-scale FSPs such as series “Car”, “Rec”, “Hel” the solution accuracy of SGA is better than that of NEH, and the best value for multiple times can reach the known optimal. However, solution accuracy of SGA has no obvious advantage over NEH heuristic algorithm. Both the performance of the best optimum and the average optimum on large-scale FSPs are not good. Except for a few series of problems, the gap ratio of solutions obtained by NEH is less than 5%. Either the average solution gap ratio or the optimal gap ratio, NEH-SGA performs best.

As Table 3 and Fig. 8 demonstrate, the solving deviation of NEH heuristic algorithm is relatively stable, which fluctuates around 2% for most instances, except for individual instances (Taillard 50 × 20), which are higher, reaching 6%. The accuracy of SGA is not much different from that of NEH on small-scale instances, or even better than that of NEH on individual instances, but it is the worst on large-scale instances. Compared with the previous two algorithms, HMSA^17^ has a large improvement in accuracy and is relatively stable. The solving deviation of HMSA can be controlled below 1% on average. In general, the proposed NEH-NGA has some improvements compared with HMSA. The known optimal solution can be reached on some instances, which may be due to the lucky commonality of population evolution algorithms.

In view of the appropriateness of Gantt chart color expression and operation number expression, we selected “Rec11” with 20 × 10 scale and “Ta31” with 50 × 5 scale as demonstration instances.

**Figure 9:**
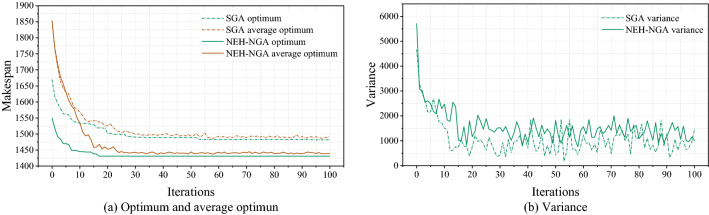
The population evolutionary process of “Rec11”.

**Figure 10:**
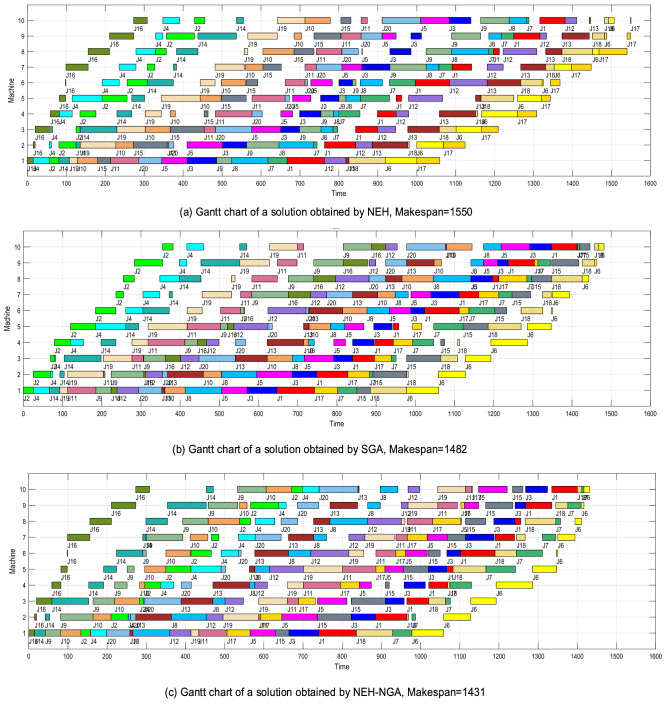
Gantt charts of different solutions to “Rec11” obtained by NEH, SGA and NEH-SGA.

Figure 9 shows the population evolutionary process of “Rec11”. As shown in Fig. 9a, in iteration 0, the optimum of SGA is 1669 and NEH-SGA is 1550. At the 53th iteration, SGA get the optimal solution 1482 and at the 17th iteration, NEH-SGA get the optimal solution 1431. We can see from Table 2 that 1431 is the known best solution to this instance. The above differences suggest that, using NEH to optimize the initial population and applying three crossover operators to enhance the genetic efficiency are resultful. The number of solutions to “Rec11” is 20! ≈ 2.43 × 10^18^ and it’s really a quite lager number. However, NEH-NGA can find the optimal one precisely that proves the search ability of the algorithm. While Fig. 10 shows the Gantt charts of different solutions to “Rec11” obtained by NEH(Makespan = 1550), SGA(Makespan = 1482) and NEH-SGA(Makespan = 1431). Obviously, NEH-SGA has the highest solution accuracy.

As shown in Fig. 9b, at the beginning of iteration, especially in the first five iterations, the population individual variance generated by SGA and NEH-NGA algorithms is basically the same, which reflects the absolute randomness of meta-heuristic algorithm. As the iteration progresses, selection mechanism makes individuals gather and population diversity decrease, resulting in smaller variance. From the 5th iteration to the 10th iteration, the variance difference between the two algorithms appeared. The rapid decline of SGA indicates that individual aggregation may tend to prematurity obviously, which can also be seen in Fig. 9a, that is, the difference between the average value and the optimal value is not large. At the same stage, the variance of NEH-SGA is still at a high level, and the difference between average value and the optimal value is also large. At the end of iteration, both the algorithms converge, and the variance of NEH-SGA is still larger than that of SGA. The above differences suggest that, applying niche mechanism to control the population distribution and maintain population diversity is resultful.

**Figure 11:**
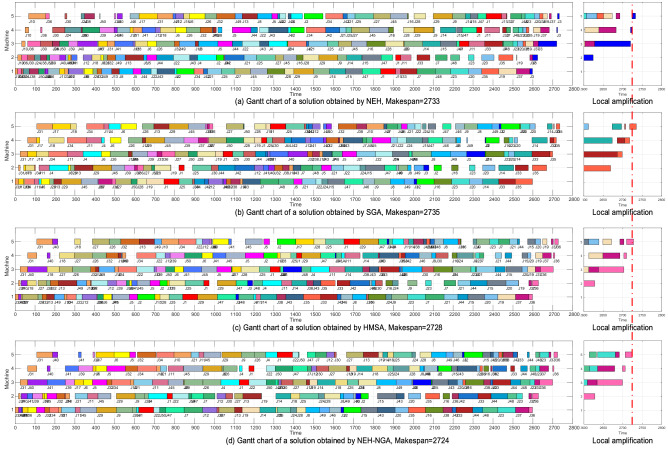
Gantt charts of different solutions to “Ta31” obtained by NEH, SGA, HMSA and NEH-SGA.

Figure 11 shows the Gantt charts of different solutions to “Ta31” obtained by NEH(Makespan = 2733), SGA(Makespan = 2735), HMSA(Makespan = 2728) and NEH-SGA(Makespan = 2724). Obviously, NEH-SGA has the highest solution accuracy. The job sequence of the solution are as follows:

NEH: 10-36-30-24-38-50-39-40-46-17-31-41-12-18-6-26-32-49-13-8-5-44-22-43-4-2-34-42-21-25-27-45-16-28-29-9-14-15-47-1-11-33-7-48-23-20-35-19-37-3.
SGA: 31-17-18-34-11-4-6-26-13-29-45-39-37-36-27-50-28-19-1-25-30-44-42-12-41-40-32–38-10-43-7-48-5-21-22-24-15-47-46-9-8-49-3-2-16-23-20-14-33-35.
HMSA: 31-40-18-27-26-32-13-49-10-34-22-12-39-50-6-41-45-5-2-17-28-25-1-29-47-3-48-4-11-14-38-43-35-33-42-46-8-30-16-24-9-23-7-21-44-15-20-19-37-36.
NEH-SGA: 31-40-41-39-17-6-5-32-34-10-21-11-45-29-9-26-4-1-22-50-47-7-12-30-27-13-19-14-18-25-24-28-8-49-46-3-2-15-43-20-35-16-38-42-33-44-48-23-37-36.

**Figure 12:**
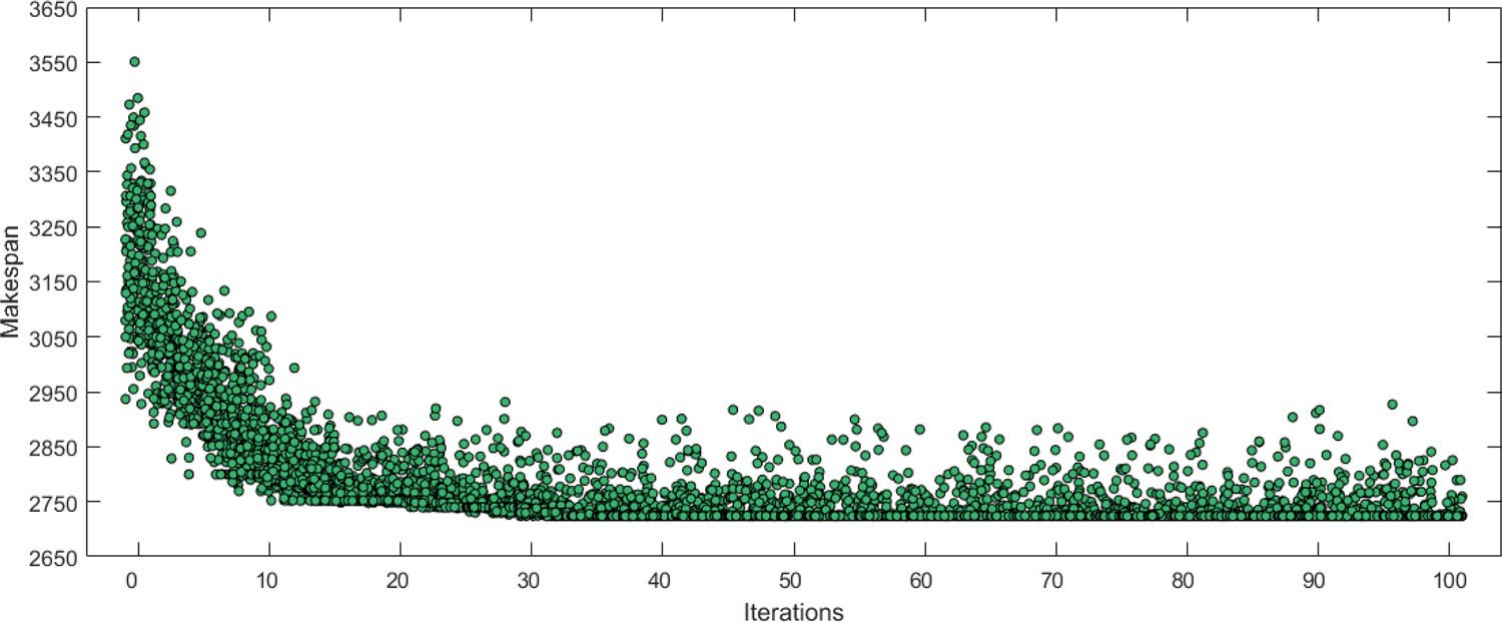
Individual distribution map of NEH-SGA on “Ta31”.

Figure 12 shows the individual distribution map of NEH-SGA on “Ta31″. As can be seen from the figure, the optimal solution appears for the first time in the 30th generation and then the population tends to converge. Since the mutation probability (*Pm*) is set at 0.1, this means that about 10% of individuals in per generation will have the mutation, and the reaction shown in distribution map as the jumping point. The mutation rule is not as random as the initial population, that is, the jumping point is not too far away from the optimal solution.

## Conclusion

In this paper, the flow shop scheduling problem was studied. In the aspect of basic research, the spatial distribution of FSP solutions is studied, and common methods for solving NP-hard problems such as FSPs are understood. Aiming at the defects of current methods in solving FSPs, a NEH-NGA algorithm with higher solving accuracy is proposed.

1. NEH is the most effective heuristic algorithm in solving FSP. This paper uses the approach of taking NEH optimized individual as the initial solution of the evolutionary algorithm to improve the search performance of the evolutionary algorithm.
2. In order to ensure that the effective features in the genetic algorithm can be better inherited to the next generation, a single genetic operator is abandoned, and three genetic operators with different performance characteristics are mixed to use.
3. In view of the advantages of niche in solving multi-peak function optimization problems, a niche idea was proposed and introduce it into GA to slow down the premature phenomenon of GA in solving large-scale FSPs.

The results of compared with NEH heuristic algorithm and SGA evolutionary metaheuristic algorithm after testing on 101 FSP benchmark instances show that the solution accuracy has been significantly improved.

Future works:

1. Considering that there is still a gap between the current optimum and the known optimal solution, an interesting subject for future researches will be the investigation of this problem by the idle time of machine to develop a heuristic local search algorithm and combined with the present global search to further improve the solution accuracy.
2. Considering the machine failure and maintenance during the production, how to deal with the post prognostic decision making in order to improve system safety and avoid downtime and inopportune maintenance spending will be another working topic.

## Data Availability

All data generated or analyzed during this study are included in this paper.
